# Neuromodulation Therapies for Depression: Current Status and Future Directions

**DOI:** 10.31083/AP53313

**Published:** 2026-10-10

**Authors:** Yuki Matsuda, Kenji Sakuma, Shun Igarashi, Daisuke Hayashi, Shinsuke Kito, Taro Kishi

**Affiliations:** ^1^Department of Development and Education of Clinical Research, Fujita Health University School of Medicine, 470-1192 Toyoake, Aichi, Japan; ^2^Department of Psychiatry, The Jikei University School of Medicine, 105-8461 Minato-ku, Tokyo, Japan; ^3^Department of Psychiatry, Fujita Health University School of Medicine, 470-1192 Toyoake, Aichi, Japan; ^4^Department of Psychiatry, National Center Hospital, National Center of Neurology and Psychiatry, 187-8551 Kodaira-shi, Tokyo, Japan

**Keywords:** difficult-to-treat depression, electroconvulsive therapy, magnetic seizure therapy, repetitive transcranial magnetic stimulation, theta-burst stimulation, treatment-resistant depression

## Abstract

Major depressive disorder is a leading cause of disability worldwide, and many patients fail to achieve sustained remission with first-line pharmacotherapy or psychotherapy. This unmet need has traditionally been framed as treatment-resistant depression (TRD), defined by an inadequate response to antidepressant treatment. Within this context, neuromodulation has assumed an increasingly important role by providing mechanistically distinct interventions when medication-based approaches are insufficient, poorly tolerated, or too slow. In this expert opinion article, we present a targeted, clinically oriented review of two established neuromodulation therapies for depression, electroconvulsive therapy (ECT) and transcranial magnetic stimulation (TMS), encompassing conventional repetitive TMS (rTMS), deep TMS, and theta-burst stimulation (TBS), as well as magnetic seizure therapy (MST), the most clinically advanced investigational convulsive modality. ECT remains the benchmark treatment for severe, urgent, psychotic, or otherwise refractory depression due to its rapid and robust efficacy, although its use is limited by anesthesia requirements, cognitive adverse effects, and associated stigma. TMS has the strongest evidence base among nonconvulsive neuromodulation treatments, with pivotal sham-controlled multicenter trials supporting conventional rTMS and deep TMS, while TBS protocols are improving feasibility, access, and scalability. MST is conceptually appealing as a more focal convulsive treatment designed to preserve antidepressant efficacy while reducing cognitive burden; recent Confirmatory Efficacy and Safety Trial of Magnetic Seizure Therapy for Depression (CREST-MST) data have strengthened its evidence base, but its routine clinical role remains to be defined through regulatory approval, durability data, and implementation evidence. We argue that future progress will depend not only on the development of new stimulation protocols but also on improved clinical sequencing, patient selection, and maintenance strategies that help sustain acute gains. The concept of difficult-to-treat depression (DTD) broadens the clinical framework beyond antidepressant nonresponse to include persistent burden, partial response, tolerability constraints, functional impairment, and patient-centered goals. This perspective may inform earlier and more strategic use of neuromodulation, although most randomized controlled trials remain focused on TRD. Further studies are needed to determine whether DTD-based approaches improve real-world outcomes. Advances are likely to arise from integrating protocol refinement, individualized targeting, scalable home-based approaches, depression-relevant extensions such as bipolar depression, and durable maintenance strategies rather than identifying a single universally superior modality.

## Main Points

1. Electroconvulsive therapy (ECT) remains the benchmark neuromodulation treatment for severe, urgent, psychotic, or highly refractory depression.

2. Transcranial magnetic stimulation (TMS) is the leading nonconvulsive neuromodulation platform for depression, with robust evidence for conventional repetitive TMS (rTMS) and deep TMS; intermittent theta-burst stimulation (iTBS) provides a shorter and more scalable protocol.

3. Magnetic seizure therapy (MST) is the most clinically advanced investigational convulsive neuromodulation treatment, with recent noninferiority evidence versus right unilateral ultrabrief-pulse ECT but an unresolved role in routine clinical care.

4. As most randomized controlled trials (RCTs) to date have focused on treatment-resistant depression (TRD), dedicated studies are needed to define the role of neuromodulation within a difficult-to-treat depression (DTD) framework in real-world care.

5. Future progress will depend not only on new technologies, but also on protocol optimization, biomarker-informed targeting, scalable home-based approaches, depression-relevant extensions, and maintenance strategies.

## 1. Introduction

Major depressive disorder (MDD) is highly prevalent, disabling, and associated with substantial societal costs, and many patients do not achieve adequate benefit from first-line treatments. Despite appropriate pharmacotherapy and psychotherapy, a clinically important subgroup continues to experience persistent depressive symptoms, functional impairment, suicidality, and repeated treatment failure [1]. This unmet need has traditionally been conceptualized as treatment-resistant depression (TRD), although no universally accepted definition exists. Regulatory definitions from the US Food and Drug Administration (FDA) and the European Medicines Agency typically require inadequate response to at least two antidepressant regimens of adequate dose and duration with confirmed adherence; however, this binary framework fails to fully capture clinical complexity, including partial response, tolerability constraints, partial improvement, and patient-centered outcomes [1].

The concept of difficult-to-treat depression (DTD) has therefore emerged as a broader clinical framework. An international consensus statement emphasizes that DTD reflects the persistence of significant disease burden despite appropriate, sequential, and individualized treatment efforts, rather than simply the number of failed interventions [2]. Notably, the current evidence base for neuromodulation therapies is derived largely from studies in TRD, with limited randomized controlled trial (RCT) data specifically addressing DTD [1,2,3]. Accordingly, this expert opinion article first reviews neuromodulation therapies based on TRD-focused evidence, and then explores their potential role and future positioning in DTD in a dedicated section.

While this perspective is highly relevant to neuromodulation, most RCTs to date have been conducted in patients meeting strict TRD criteria. In real-world practice, however, candidacy for neuromodulation is not determined solely by formal TRD thresholds, but also by clinical considerations such as required speed of response, cognitive profile, prior treatment response, and individualized therapeutic goals. These factors may favor neuromodulation over further pharmacological iteration. Therefore, further studies are needed to clarify how a DTD-based framework can best inform neuromodulation treatment selection and sequencing.

Neuromodulation occupies a distinct role in the management of TRD by targeting neural circuits rather than relying solely on systemic pharmacology. Among available or clinically advanced strategies, three modalities are especially important. Electroconvulsive therapy (ECT) remains the most established convulsive treatment and the benchmark for rapid, robust antidepressant efficacy. Transcranial magnetic stimulation (TMS), including conventional repetitive protocols, deep TMS, and intermittent theta-burst stimulation (iTBS), is the leading nonconvulsive neuromodulation therapy for TRD and continues to evolve toward shorter, more scalable protocols.

Magnetic seizure therapy (MST), although still investigational and not yet established in routine clinical practice, is the most clinically advanced effort to develop a more focal convulsive therapy. It aims to preserve the rapid and robust antidepressant efficacy of ECT while reducing cognitive adverse effects through more spatially restricted seizure induction. However, MST retains important limitations, including the need for anesthesia, specialized equipment, and confirmatory evidence before routine clinical adoption.

The key clinical and mechanistic differences among ECT, conventional rTMS, deep TMS, iTBS, and MST are summarized in Table 1. In this expert opinion article, we review the current status of these modalities and discuss future directions that may refine the sequencing of neuromodulation in TRD management. Throughout, TMS is used as the umbrella term for the broader transcranial magnetic stimulation platform. When referring specifically to standard repetitive protocols, we use conventional rTMS (e.g., high-frequency left dorsolateral prefrontal cortex [DLPFC] rTMS or low-frequency right DLPFC rTMS). Theta-burst stimulation (TBS), including iTBS, is discussed separately as a patterned TMS protocol, and deep TMS is specified explicitly when relevant.

**Table 1.** **Clinical and mechanistic features of ECT, conventional rTMS, deep TMS, iTBS, and MST in treatment-resistant depression**.

Feature	ECT	Conventional rTMS	Deep TMS	iTBS	MST
Clinical and regulatory status	• Status: Established/reference standard • Type: Convulsive (anesthesia required)	• Status: Established (FDA-cleared in the United States; clinically established/CE-marked in Europe) • Type: Nonconvulsive focal stimulation (outpatient, no anesthesia required)	• Status: Established (FDA-cleared in the United States; clinically established/CE-marked in Europe) • Type: Nonconvulsive stimulation targeting broader and relatively deeper prefrontal circuits (outpatient, no anesthesia required)	• Status: Established as a shorter TMS protocol (FDA-cleared in the United States; clinically established/CE-marked in Europe) • Type: Nonconvulsive focal patterned stimulation (outpatient, no anesthesia required)	• Status: Investigational (clinically advanced) • Type: Convulsive magnetic induction (under anesthesia)
Putative mechanism of action	• Broad neurobiological reset (monoamines, BDNF, neuroplasticity) • Normalization of structural and functional brain abnormalities associated with severe depression	• Focal neuronal depolarization via electromagnetic induction (Faraday’s law) • Frequency-dependent neuroplasticity • Downstream modulation of mood-regulating circuits (e.g., the subgenual anterior cingulate cortex)	• Magnetic stimulation engages broader and relatively deeper prefrontal circuits than conventional figure-of-eight coil approaches • Downstream modulation of mood-regulating circuits with broader target engagement	• Burst-pattern stimulation induces potent LTP-like plasticity in a shorter time frame • Downstream modulation of mood-regulating circuits similar to conventional rTMS, but with greater time efficiency	• Induction of a generalized therapeutic seizure via focal cortical magnetic stimulation • Relative sparing of deeper medial temporal and hippocampal structures compared with ECT
Clinical role and profile	• Target population: Patients with severe, urgent, psychotic, nutritionally compromised, or highly treatment-resistant depression (TRD) • Efficacy: Response, 70–80%; remission, 50–60% • Advantage: Most robust and rapid acute efficacy	• Target population: Patients with outpatient TRD when a nonconvulsive, cognitively sparing option is preferred • Efficacy: Response, 40%–50%; remission, 30% • Advantage: Established outpatient platform with the broadest conventional evidence base	• Target population: Patients with outpatient depression/TRD when broader target engagement within the TMS platform is desired • Efficacy: Sham-controlled efficacy has been demonstrated; evidence supports effectiveness and tolerability, although the literature is less extensive than that for conventional rTMS • Advantage: Broadens target engagement while preserving outpatient feasibility and tolerability	• Target population: Patients with outpatient TRD when treatment efficiency, feasibility, and scalability are especially important • Efficacy: Noninferior to standard high-frequency rTMS in a clinical trial • Advantage: Markedly shorter session duration while preserving antidepressant efficacy	• Target population: Patients requiring convulsive therapy but at high risk for cognitive adverse effects from ECT • Efficacy: Noninferior remission compared with right unilateral ultrabrief-pulse (RUL-UB) ECT in the CREST-MST trial • Advantage: Potential to reduce cognitive burden while maintaining the benefits of convulsive therapy
Main limitations & adverse effects	• Burden: High (anesthesia, intensive treatment setting) • AEs: Postictal disorientation; anterograde and retrograde autobiographical memory loss • Limitation: High stigma, the requirement for anesthesia, and the need for continuation or maintenance treatment planning	• Burden: Low (outpatient) • AEs: Transient scalp discomfort and headache • Limitation: Very rare seizure risk (<0.1%); may be less effective in the most acute or severe cases	• Burden: Low (outpatient) • AEs: Generally similar to those of other TMS modalities, including scalp discomfort and headache • Limitation: Smaller and less extensively characterized evidence base than conventional rTMS	• Burden: Low (outpatient) • AEs: Transient scalp discomfort and headache • Limitation: Durability, optimal dosing, and maintenance strategies are less well characterized than those for conventional high-frequency rTMS	• Burden: High (requires anesthesia and specialized equipment) • AEs: Preliminary evidence suggests a more favorable cognitive profile than ECT, but long-term safety and durability remain insufficiently characterized • Limitation: Insufficient evidence for routine clinical use; lack of standardized treatment parameters

Abbreviations: AEs, adverse effects; BDNF, brain-derived neurotrophic factor; CE, *Conformité Européenne*; CREST-MST, *Confirmatory Efficacy and Safety Trial of Magnetic Seizure Therapy for Depression*; ECT, electroconvulsive therapy; FDA, United States Food and Drug Administration; iTBS, intermittent theta-burst stimulation; LTP, long-term potentiation; MST, magnetic seizure therapy; rTMS, repetitive transcranial magnetic stimulation; RUL-UB ECT, right unilateral ultrabrief-pulse electroconvulsive therapy; TMS, transcranial magnetic stimulation; TRD, treatment-resistant depression. Regulatory clearance, including FDA clearance or CE marking where applicable, approved indications, device labeling, protocol specifications, reimbursement, and clinical availability vary by country or region, device, indication, and local practice environment. Therefore, this table summarizes the broad clinical positioning of these modalities rather than providing a jurisdiction-specific summary of regulatory status or reimbursement.

## 2. Materials and Methods

This article was prepared as a targeted narrative Expert Opinion rather than as a systematic review or meta-analysis. Therefore, we did not register a review protocol or apply prespecified systematic eligibility criteria, PRISMA-based screening, record-by-record study selection, or a formal risk-of-bias assessment. The literature search was intended to support a clinically oriented synthesis of the major evidence relevant to neuromodulation therapies for depression, particularly TRD, and to inform their potential future positioning within a DTD framework. This narrative review prioritized evidence from higher-level study designs, including meta-analyses, systematic reviews, and randomized controlled trials, in hierarchical order, supplemented by clinical practice guidelines, large observational studies, and selected emerging evidence when directly relevant to the scope of this Expert Opinion.

This expert opinion article reviews the utility of neuromodulation treatments in TRD, while considering the potential conceptual relevance of DTD for future treatment sequencing. We first identified neuromodulation therapies established within current regulatory and clinical frameworks in the United States and Europe for depressive disorders. This approach identified ECT and TMS, including conventional high-frequency rTMS, deep TMS, and iTBS, as established clinical modalities. To address future directions, we also considered MST, accelerated and individualized TMS protocols, home-based transcranial direct current stimulation (HB-tDCS), bipolar depression, biomarker-informed targeting, and DTD-oriented treatment sequencing.

We then conducted targeted literature searches in PubMed through March 30, 2026 using the term “Depressive Disorder [MeSH]” in combination with modality-specific terms, including “electroconvulsive therapy”, “transcranial magnetic stimulation”, “repetitive transcranial magnetic stimulation”, “deep transcranial magnetic stimulation”, “theta-burst stimulation”, “intermittent theta-burst stimulation”, “magnetic seizure therapy”, “transcranial direct current stimulation”, and “home-based transcranial direct current stimulation”. Reference lists of retrieved articles were also screened to identify additional relevant studies. For the “Current Status” sections, we prioritized pivotal clinical trials, randomized controlled trials, systematic reviews, meta-analyses, and current clinical practice guidelines, where available. For the “Future Directions” sections, we additionally considered selected literature addressing accelerated and individualized TMS protocols, HB-tDCS, bipolar depression, biomarker-informed targeting, DTD-oriented treatment sequencing, and maintenance or implementation issues. For each modality, we summarize mechanisms of action, clinical trial evidence, and safety profiles. For ECT, given the absence of a modern sham-controlled phase III trial framework, we also incorporated current clinical practice guidelines and high-level reviews to contextualize its contemporary role in depression management. The search was supplemented by targeted updates incorporating pivotal evidence published during manuscript revision, including the *Confirmatory Efficacy and Safety Trial of Magnetic Seizure Therapy for Depression* (CREST-MST) trial.

## 3. Current Status of Neuromodulation Therapies

### 3.1 Electroconvulsive Therapy

Because ECT predates contemporary drug and device development frameworks, it lacks modern sham-controlled phase III trials analogous to those used for TMS; its present status is therefore best informed by long-standing clinical use, meta-analyses of relapse and continuation outcomes, and contemporary practice guidelines [4,5,6,7,8]. On this basis, ECT remains the reference-standard neuromodulation treatment for severe depression. Its clinical importance reflects not only historical precedence but also its consistent ability to produce substantial and often rapid antidepressant effects in patients with high symptom burden, psychotic features, nutritional compromise, or urgent suicidality. Recent reviews summarize response rates of approximately 70%–80% and remission rates of 50%–60% even in treatment-resistant populations, distinguishing ECT from other established neuromodulation treatments [9].

ECT is understood to act through seizure-induced modulation of multiple neurobiological systems rather than a single mechanism. Proposed mechanisms include monoaminergic effects, neuroplastic changes, immunomodulation, and normalization of structural and functional brain abnormalities associated with severe depression [9]. Clinically, this differentiates ECT from focal neuromodulation approaches in both intensity and breadth of circuit engagement. This broader network effect likely underlies its high efficacy in biologically severe depression, while also contributing to its cognitive adverse effect profile.

In practice, ECT is typically introduced after inadequate response to multiple antidepressant and augmentation strategies, and often functions as a last-step option in non-urgent TRD. However, in severe, psychotic, nutritionally compromised, or otherwise urgent presentations, it may be used earlier, as the key consideration is not only treatment efficacy but also the feasibility of waiting for slower-acting interventions [1,4,5]. Thus, even when ECT is considered relatively late in the treatment course, its use should be guided by a careful appraisal of expected remission, treatment burden, anesthesia requirements, and cognitive risk. The principal neurocognitive adverse effects include postictal disorientation, anterograde memory impairment affecting new learning, and retrograde autobiographical memory loss, the latter often being the most distressing [6,10]. Although group-level objective cognitive performance usually returns toward baseline within days to weeks and new learning often normalizes within approximately 2 weeks, subjective memory complaints may persist in a subset of patients [10]. Modern practice seeks to mitigate these risks through refinements in pulse width, electrode placement, dose titration, and serial cognitive monitoring [5,6].

A second challenge is durability. Despite strong acute efficacy, relapse is common without structured continuation strategies. A meta-analysis revealed relapse rates of 37.7% at 6 months and 51.1% at 12 months following successful ECT despite continuation of pharmacotherapy, with the highest risk observed within the first 6 months [7]. These findings underscore the importance of continuation and maintenance of ECT in sustaining acute clinical gains. Such protocols are typically initiated after the acute course, often at weekly intervals and then progressively spaced out based on symptom recurrence and tolerability [6,8]. Although the evidence base remains limited and heterogeneous, recent meta-analytic evidence indicates that continuation ECT combined with pharmacotherapy reduces relapse compared with pharmacotherapy alone, while large real-world cohort studies suggest lower rehospitalization rates with continuation or maintenance ECT following an acute ECT course [8,11]. Accordingly, ECT should be conceptualized not as a standalone endpoint but as the beginning of a relapse-prevention strategy. Future advances are likely to focus on improving acceptability, cognitive safety, and maintenance strategies rather than on demonstrating efficacy.

### 3.2 Conventional Repetitive Transcranial Magnetic Stimulation

Among nonconvulsive neuromodulation treatments, conventional repetitive transcranial magnetic stimulation (rTMS) has the most robust contemporary evidence base and the clearest translational path from clinical trials to routine practice. Its central advantage is the ability to modulate dysfunctional prefrontal-limbic networks without anesthesia and without the persistent cognitive adverse effects associated with ECT. This profile makes rTMS particularly suitable for outpatients with TRD who prioritize cognitive preservation, functional continuity, or nonpharmacological strategies. The Canadian Network for Mood and Anxiety Treatments now includes several TMS protocols among recommended treatments for depression, including high-frequency left DLPFC rTMS, low-frequency right DLPFC rTMS, and left-sided iTBS as first-line neuromodulation options [5].

Mechanistically, TMS is grounded in Faraday’s law of electromagnetic induction: a rapidly changing magnetic field generated by the coil induces an electric field in superficial cortical tissue, leading to neuronal depolarization [12]. Repetitive stimulation produces frequency- and pattern-dependent long-term potentiation-like (LTP-like) or long-term depression-like (LTD-like) neuroplastic effects [13]. In depression, therapeutic effects are thought to arise not only from local excitation of the DLPFC but also from downstream effects on distributed mood-regulating circuits, including functionally connected subcortical and paralimbic regions such as the subgenual anterior cingulate cortex [13,14]. This network-level framework underpins its role as a circuit-based treatment for depression [13].

The clinical status of conventional rTMS is anchored on pivotal sham-controlled trials. In the multicenter study by O’Reardon et al. [15], active left prefrontal TMS showed superior efficacy to sham in patients with TRD and was well tolerated, establishing TMS as a credible antidepressant modality. The subsequent OPT-TMS trial led by George et al. [16] confirmed statistically significant and clinically meaningful benefits of daily left prefrontal rTMS monotherapy versus sham in a large multicenter design. Together, these trials established the reproducibility of antidepressant effects across controlled, multisite settings.

The conventional rTMS literature also indicates that efficacy is not uniform across populations or settings. The veterans trial by Yesavage et al. [17] is an important negative multicenter study: despite substantial overall remission, active rTMS did not demonstrate a statistically significant advantage over sham treatment. This finding underscores that the observed efficacy of rTMS is influenced by patient selection, baseline severity and illness chronicity, concomitant treatments, expectancy effects, sham design, stimulation parameters, targeting methods, and implementation context. No formal assessment of publication bias was performed because it was beyond the scope of this narrative review; however, findings from smaller positive studies should be interpreted in the context of this heterogeneity and the mixed evidence from controlled trials. In clinical practice, the TMS platform is best understood as a family of protocols applied to heterogeneous patients, with effectiveness partly dependent on appropriate matching of protocol to illness stage and the quality of implementation.

From a comparative standpoint, the TMS platform is best understood as the primary outpatient neuromodulation therapy for TRD. It is generally more acceptable than ECT, does not require anesthesia, and has a favorable neurocognitive profile, although it may be less effective than ECT in severe, urgent, psychotic, or highly refractory states. In broad clinical terms, conventional rTMS achieves response rates of approximately 40%–50% and remission rates around 30%, lower than those typically reported for ECT but clinically meaningful given its outpatient feasibility and cognitive tolerability [1,5]. Common adverse events include scalp discomfort or pain at the stimulation site and headache, while seizure is a very rare but important serious event even when safety protocols are followed [18]. These estimates should not be interpreted as pooled or directly comparable efficacy estimates but rather as approximate clinical ranges because of differences across studies in patient severity, definitions of treatment resistance and clinical outcomes, stimulation or electrode/coil parameters, comparator conditions, and follow-up duration. Collectively, these considerations support a more precise positioning of the TMS platform, not as a less effective alternative to ECT, but as the leading scalable neuromodulation option for patients whose depression is substantial enough to warrant circuit-based intervention but not so acute that it requires the speed and potency of ECT.

### 3.3 Deep Transcranial Magnetic Stimulation

Deep transcranial magnetic stimulation (deep TMS) represents an important extension of the broader TMS platform because it is designed to stimulate broader and deeper prefrontal circuits compared with conventional figure-of-eight coil approaches. Its rationale is not to replace conventional rTMS, but to broaden target engagement while preserving the nonconvulsive, outpatient advantages and favorable tolerability of TMS [19,20].

The pivotal multicenter double-blind RCT by Levkovitz et al. [19] demonstrated significantly greater improvement in depressive symptoms, along with higher response and remission rates, with deep TMS compared with sham stimulation in TRD. This study provided the key sham-controlled evidence supporting regulatory clearance and clinical adoption of deep TMS in TRD. Consistent with these findings, a systematic review and meta-analysis by Hung et al. [21] concluded that deep TMS is effective and well tolerated, although the evidence base remains relatively limited.

From a comparative standpoint, deep TMS is best viewed as a variant of the TMS platform rather than as a distinct therapeutic class. A recent review comparing rTMS and deep TMS in TRD found both approaches to be effective and safe, with no clear evidence of major differences in efficacy based on the limited direct-comparison data [20]. Accordingly, the present role of deep TMS is to broaden target engagement and therapeutic options within TMS, rather than to provide a clearly superior alternative to conventional rTMS.

### 3.4 Intermittent Theta-Burst Stimulation

Intermittent theta-burst stimulation (iTBS) represents the most important refinement of the TMS platform, preserving antidepressant efficacy while markedly reducing treatment time. In this article, standard iTBS refers to the brief outpatient TBS protocol typically delivered once daily over a conventional treatment course, whereas accelerated intermittent theta-burst stimulation (aiTBS) refers to protocols that deliver multiple iTBS sessions per day over a compressed treatment schedule. Our pairwise meta-analysis found that iTBS has antidepressant efficacy comparable to that of high-frequency rTMS in TRD. The substantially shorter treatment duration of iTBS may offer practical advantages for patients and healthcare providers [22].

This equivalence is anchored by the THREE-D trial, a landmark non-inferiority trial demonstrating that iTBS is non-inferior to standard high-frequency rTMS for TRD while reducing treatment time from approximately 37.5 minutes to about 3 minutes per session [23]. This finding was clinically important not only because it supported the efficacy of iTBS, but also because it changed the logistical profile of TMS in routine psychiatric care. By substantially shortening session duration, iTBS improved the feasibility, throughput, and scalability of outpatient neuromodulation delivery.

These practical advantages matter because the real-world impact of neuromodulation depends not only on efficacy but also on deliverability and accessibility in busy clinical settings. iTBS may therefore be especially valuable where time burden limits access to care. However, key questions remain regarding durability, optimal dosing, accelerated schedules, and maintenance planning.

## 4. Future Directions and Emerging Prospects

Advancing neuromodulation for depression will require a conceptual shift alongside technical innovation. The field has largely been structured around the binary construct of TRD, whereas a DTD framework offers a more clinically informative approach to treatment sequencing by emphasizing that many patients live with meaningful residual burden despite partial response. In this context, the central question is not simply how many antidepressants have failed, but which next intervention is most likely to improve symptoms, functioning, tolerability, and long-term recovery for the individual patient [1,2,3]. Framed in this way, future progress in neuromodulation can be organized around four depression-focused priorities: maturation of next-generation convulsive therapy, refinement and personalization of stimulation protocols, scalable home-based adjunctive approaches; and depression-relevant extensions, including bipolar depression. These future directions are subsequently integrated with the DTD framework as a pragmatic approach to treatment sequencing, maintenance planning, and clinical implementation.

### 4.1 Magnetic Seizure Therapy

MST is the most clinically advanced investigational convulsive neuromodulation treatment for depression. It occupies a deliberate intermediate position between ECT and TMS. Like ECT, it is administered under anesthesia and induces a therapeutic seizure; like TMS, it uses magnetic rather than electrical stimulation to initiate a generalized seizure from more focal cortical stimulation, thereby reducing unintended effects on brain regions involved in cognitive function. The central objective is to retain the robust antidepressant efficacy of convulsive therapy while reducing the cognitive burden that constrains wider acceptance of ECT [24,25,26].

Current evidence is promising but insufficient for routine clinical adoption. A Cochrane review by Jiang et al. [25] identified MST as a plausible alternative for TRD but emphasized that the evidence base remains small and uncertain. A comparative meta-analysis similarly suggested that MST may provide a more favorable cognitive profile than ECT while maintaining meaningful antidepressant effects, although the underlying trials were limited by small sample sizes and methodological heterogeneity [26]. Data on durability are sparse; however, Tang et al. [24] reported that approximately two-thirds of patients maintained symptomatic improvement over 6 months of continuation MST without evident adverse cognitive effects.

The recently published CREST-MST trial has substantially strengthened the evidence base for MST. In this multicenter, randomized, double-blind, parallel-group, noninferiority trial conducted in Canada and the United States, 239 participants with MDD were randomly assigned to receive either MST or right unilateral ultrabrief-pulse ECT (RUL-UB ECT) and were treated until remission, withdrawal, or completion of up to 21 treatment sessions. MST met the prespecified noninferiority criterion for remission (remission rates: MST, 22.5%; RUL-UB ECT, 27.8%) and was associated with less worsening of autobiographical memory than RUL-UB ECT (2.7% vs. 17.3%) [27]. These findings support MST as a clinically important investigational convulsive therapy, particularly for patients in whom cognitive adverse effects represent a major barrier to ECT. However, the trial compared MST with a single optimized ECT approach rather than the full range of ECT approaches, and enrollment ended before the intended sample size was reached. In addition, long-term durability, maintenance strategies, parameter standardization, regulatory evaluation, and implementation pathways remain insufficiently established. MST should therefore continue to be regarded as a promising investigational modality rather than a current standard of care [5,27].

### 4.2 Innovations in Stimulation Protocols: Stanford Neuromodulation Therapy

Accelerated intermittent TBS (aiTBS) represents a key evolution of the TMS platform by extending the time efficiency of iTBS to intensive, compressed treatment schedules. The most prominent example is Stanford Neuromodulation Therapy (SNT), which delivers multiple daily individualized iTBS sessions with a large cumulative pulse dose over several days. A double-blind randomized trial by Cole et al. [28] generated substantial interest by demonstrating the potential for rapid remission in selected TRD populations using intensive, target-guided stimulation.

Interpretation of SNT requires recognizing that its apparent efficacy likely reflects more than protocol acceleration alone. In addition to compressed delivery and dose intensification, SNT incorporates individualized targeting based on circuit-level models, particularly functional connectivity with regions such as the subgenual anterior cingulate cortex. It therefore represents a convergence of accelerated scheduling, higher cumulative dose, and precision targeting rather than a simple increase in treatment intensity.

SNT also suggests that future progress in TMS will likely come not from finding a single best protocol for all patients, but from better matching stimulation parameters and targets to individual patients. Consistent with this, Benster et al. [29] identified key priorities including improved target selection, individualized protocol design, integration of MRI or EEG markers, and clarification of the difference between delivered dose and biologically received dose. In practical terms, the next phase of TMS development will depend less on expanding protocol variants and more on better matching across patient phenotypes, circuit dysfunction, stimulation parameters, and maintenance approaches. A parallel priority is biomarker-informed stratification. Rather than relying solely on post hoc correlations in heterogeneous samples, future studies should prospectively identify and validate biomarkers that reflect the principal pathophysiological mechanisms underlying predefined depressive subtypes and clinically relevant symptom dimensions. Candidate biomarkers include DLPFC–subgenual anterior cingulate functional connectivity and MRI- or EEG-based measures of network engagement, cortical excitability, and neuroplasticity [28,29]. These biomarkers should be evaluated as predictors, moderators, or mediators of response to specific neuromodulation protocols, thereby linking pathophysiology to protocol selection, therapeutic efficiency, durability, and maintenance requirements. At present, biomarker-guided neuromodulation remains investigational and should complement, rather than replace, comprehensive clinical assessment and guideline-supported indications.

### 4.3 Home-Based Transcranial Direct Current Stimulation

Home-based transcranial direct current stimulation (HB-tDCS) has emerged as a potentially scalable adjunctive, nonconvulsive neuromodulation approach for MDD. Its advantages include accessibility, a low procedural burden, and compatibility with longitudinal or maintenance-oriented care models. According to a recent systematic review and meta-analysis, HB-tDCS improved depressive symptoms compared with sham stimulation, with low heterogeneity across the included studies [30]. However, the evidence base remains preliminary compared with that for clinic-based neuromodulation modalities. Therefore, the available HB-tDCS data should not be interpreted as demonstrating efficacy comparable to that of antidepressants, iTBS, or other active treatments in the absence of adequately powered head-to-head trials. Further high-quality research is needed to determine the optimal stimulation parameters, adherence and remote-monitoring requirements, long-term durability, safety in real-world home settings, and the patient populations most likely to benefit from HB-tDCS. Accordingly, HB-tDCS is discussed here as an emerging future direction rather than being included in Table 1, which focuses on established or clinically advanced modalities central to current TRD-oriented neuromodulation sequencing.

### 4.4 Bipolar Depression as a Depression-Relevant Future Direction

Although the present review focuses on unipolar MDD and TRD, bipolar depression represents a depression-relevant condition in which neuromodulation strategies are being actively investigated. In bipolar depression, a systematic review incorporating pairwise and network meta-analyses suggested that TMS-based interventions were superior to sham stimulation for treatment response and improvement in depressive symptoms. Within the network meta-analysis, bilateral rTMS outperformed sham, whereas left-sided deep TMS and left-sided iTBS demonstrated only preliminary signals of benefit [31]. A recent randomized controlled trial by Sheline et al. [32] further demonstrated that accelerated iTBS was more effective than sham stimulation in reducing depressive symptoms in treatment-refractory bipolar depression. These findings suggest that selected TMS-based protocols may have potential applications beyond unipolar MDD. However, bipolar depression differs clinically and biologically from unipolar depression; therefore, these findings should not be interpreted as supporting the routine extrapolation of neuromodulation protocols established for unipolar TRD. Future studies should clarify appropriate patient selection, the risk of mood switching, long-term durability, maintenance strategies, and integration with mood-stabilizing pharmacotherapy.

### 4.5 Position of Neuromodulation in Difficult-to-Treat Depression

The concept of DTD broadens the clinical focus from simple antidepressant nonresponse to persistent disease burden despite sequential, guideline-concordant treatment. It explicitly incorporates functional impairment, tolerability constraints, partial but clinically meaningful improvement, comorbidity, and patient-centered goals [1,2,3]. Although DTD is increasingly recognized as a clinically useful framework, direct RCT evidence specifically targeting DTD remains limited [33]. Nevertheless, emerging consensus emphasizes the need to strategically sequence interventional treatments, including neuromodulation, within broader patient-centered care pathways rather than relying on repeated pharmacological iteration alone [34].

Within this framework, DTD should be viewed not as a separate evidence-based indication or formal treatment algorithm for neuromodulation but as a pragmatic decision-support framework. Its practical role is to identify circumstances in which persistent symptom burden, partial response, tolerability constraints, cognitive concerns, functional impairment, clinical urgency, prior treatment response, or patient-centered goals make continued pharmacological iteration less attractive, while evidence derived from TRD studies and guideline-supported indications remains the basis for treatment selection. Within this individualized framework, a pragmatic, nonprescriptive approach to treatment sequencing can be described in broad terms. ECT remains the preferred option when rapid and robust symptom relief is required, especially in severe or urgent presentations [4,5,6,7,8,9,10,11]; TMS, including conventional rTMS, deep TMS, and iTBS, may be preferable when outpatient feasibility and cognitive preservation are prioritized [5,15,16,17,18,19,20,21,22,23]. MST should currently be regarded as investigational and not part of routine DTD care [24,25,26,27]. Illustratively, DTD-related clinical features may influence treatment sequencing in different directions: patients with severe, psychotic, suicidal, nutritionally compromised, or otherwise urgent presentations may benefit from earlier ECT; nonurgent outpatients with persistent symptom burden, partial response, medication intolerance, and significant concerns regarding cognition or functional continuity may benefit more from conventional rTMS, deep TMS, or iTBS; and patients who require convulsive therapy but decline ECT because of concerns about memory may be candidates for MST only within future clinical practice if established or in research settings while it remains investigational. Across all modalities, acute treatment selection should be linked from the outset to continuation or maintenance planning, relapse monitoring, tolerability, and patient-centered functional goals. These positions reflect a conceptual application of the DTD framework rather than evidence of DTD-specific efficacy, and highlight several important evidence gaps:

• Randomized or pragmatic trials enrolling patients according to DTD-oriented criteria that directly evaluate neuromodulation sequencing strategies rather than single-modality efficacy alone.

• Validated clinical, neurocognitive, and biomarker predictors to guide selection among ECT, conventional rTMS, deep TMS, iTBS, and investigational MST in patients with persistent depressive burden.

• Studies evaluating continuation and maintenance strategies, relapse prevention, patient-reported and functional outcomes, acceptability, access, and cost-effectiveness within DTD-oriented care pathways [1,2,3,33,34].

### 4.6 Limitations

This article is a targeted narrative Expert Opinion rather than a systematic review or meta-analysis. Accordingly, it did not include a prespecified systematic study-selection process or a formal risk-of-bias assessment. The available evidence is heterogeneous across neuromodulation modalities, patient populations, treatment protocols, and outcome definitions; therefore, the response and remission rates summarized herein should not be interpreted as directly comparable. Furthermore, because most available studies have been conducted in patients with TRD, the proposed application of neuromodulation within a DTD framework remains conceptual and requires prospective validation.

## 5. Conclusions

Neuromodulation for depression is no longer a peripheral topic. ECT remains the benchmark intervention for severe and urgent depressive illness; TMS has become the leading scalable outpatient platform; and MST stands out as the most important investigational effort to modernize convulsive therapy. These modalities are best viewed as complementary rather than mutually exclusive, with optimal use determined by timing, illness severity, speed of response, cognitive risk, patient preference, and the broader care pathway.

The evidence base for neuromodulation remains largely grounded in TRD. However, a DTD framework offers a more clinically nuanced approach by emphasizing persistent burden, partial response, tolerability constraints, cognitive priorities, and patient-centered goals. This perspective may support earlier and more strategic use of neuromodulation, although further studies are needed to determine whether DTD-based stratification improves treatment sequencing, maintenance, and long-term outcomes.

Future progress will depend not only on technical innovation but also on clinical integration. Accelerated protocols, precision targeting, scalable home-based approaches, depression-relevant extensions such as bipolar depression, biomarker-informed treatment selection, and robust maintenance strategies are likely to define the next phase of development. If these advances are realized, neuromodulation may evolve from being a set of specialist rescue treatments into a more integrated and earlier component of precision psychiatry for depression.

## Data Availability

No new datasets were generated or analyzed for this expert opinion article.

## Abbreviations

aiTBS, accelerated intermittent theta-burst stimulation; CREST-MST, *Confirmatory Efficacy and Safety Trial of Magnetic Seizure Therapy for Depression*; DTD, difficult-to-treat depression; ECT, electroconvulsive therapy; FDA, Food and Drug Administration; HB-tDCS, home-based transcranial direct current stimulation; iTBS, intermittent theta-burst stimulation; MDD, major depressive disorder; MST, magnetic seizure therapy; RCT, randomized controlled trial; rTMS, repetitive transcranial magnetic stimulation; RUL-UB ECT, right unilateral ultrabrief-pulse electroconvulsive therapy; SNT, Stanford Neuromodulation Therapy; TBS, theta-burst stimulation; TMS, transcranial magnetic stimulation; TRD, treatment-resistant depression.
